# A Real-Time Object Detector for Autonomous Vehicles Based on YOLOv4

**DOI:** 10.1155/2021/9218137

**Published:** 2021-12-10

**Authors:** Rui Wang, Ziyue Wang, Zhengwei Xu, Chi Wang, Qiang Li, Yuxin Zhang, Hua Li

**Affiliations:** ^1^Changchun University of Science and Technology, School of Compute Science and Technology, Changchun, Jilin 130022, China; ^2^Chengdu University of Technology, Department of Geophysics, Chengdu, Sichuan 610059, China

## Abstract

Object detection is an important part of autonomous driving technology. To ensure the safe running of vehicles at high speed, real-time and accurate detection of all the objects on the road is required. How to balance the speed and accuracy of detection is a hot research topic in recent years. This paper puts forward a one-stage object detection algorithm based on YOLOv4, which improves the detection accuracy and supports real-time operation. The backbone of the algorithm doubles the stacking times of the last residual block of CSPDarkNet53. The neck of the algorithm replaces the SPP with the RFB structure, improves the PAN structure of the feature fusion module, adds the attention mechanism CBAM and CA structure to the backbone and neck structure, and finally reduces the overall width of the network to the original 3/4, so as to reduce the model parameters and improve the inference speed. Compared with YOLOv4, the algorithm in this paper improves the average accuracy on KITTI dataset by 2.06% and BDD dataset by 2.95%. When the detection accuracy is almost unchanged, the inference speed of this algorithm is increased by 9.14%, and it can detect in real time at a speed of more than 58.47 FPS.

## 1. Introduction

In recent years, deep learning has been widely applied in various fields, including computer vision [1], social services [2], and autonomous driving [3]. With the rapid development of sensors and GPU, the computing speed of deep learning algorithm is greatly accelerated, especially in the past decade, when it has been noticed that the fully autonomous vehicles might become a reality in the foreseeable future. According to the report, two-thirds of the fatal accidents every year are related to the urban traffic network [4], and the variability of autonomous driving scenes (such as cars and people in different weather, different light, and with or without occlusion) makes it particularly difficult to detect them accurately. Therefore, there are still many difficulties in the detection task.

The main task of autonomous driving is to accurately and quickly detect the vehicles, pedestrians, traffic lights, traffic signs, and other objects around the vehicles, in order to ensure the safety in driving. Generally, autonomous vehicles use various sensors, such as cameras, lidar, and radar, to detect objects [5]. Some researchers [6] detect vehicles by extracting binary images from discrete sensor arrays, and some researchers [7] have achieved good results in the detection task in bad weather through the sensing method of radar and camera information fusion. Compared with other sensors, the camera is now more accurate and more cost-effective at detecting objects. Object detection algorithm based on deep learning becomes an essential method in autonomous driving because it can achieve high detection accuracy with less computing resources.

Object detection algorithm of autonomous vehicles should satisfy the following two conditions: First, high detection accuracy of road objects is needed. Secondly, a real-time detection speed is very important for whether the detector can be used in driving. Object detection algorithms based on deep learning can be roughly divided into two categories: two-stage and one-stage. Two-stage algorithm generates region proposal in the first stage and goes on bbox regression and object classification prediction in these regions in the second stage, e.g., R-CNN [8], Fast R-CNN [9], Faster R-CNN [10], and R-FCN [11]. Two-stage algorithms usually have a high accuracy but have a relatively slow detection speed. One-stage algorithms, such as SSD [12] and YOLO [13], perform classification and regression in just one stage. These methods generally have a low accuracy but a high detection speed. In recent years, object detectors combining various optimization methods have been widely studied [14–18] in order to take advantage of both types of method. MS-CNN [14], a two-stage object detection algorithm, improves detection speed by a series of intermediate layers. RFBNet [18], a one-stage algorithm, proposes receptive filed blocks to expand the receptive field to improve accuracy. However, previous studies [14–17] can no longer satisfy the detector speed above 30 fps, one of the prerequisites for autonomous driving, when the input resolution reaches 512 × 512 or higher. This indicates that the previous schemes are incomplete in terms of the trade-off between accuracy and speed and therefore difficult to apply in the field of autonomous driving.

The problem of most object detection algorithms is that large objects are easily detected, while small objects are often ignored by the detector. It is extremely dangerous to miss pedestrians, traffic lights, and traffic signs in autonomous driving. In recent years, there are many feature fusion algorithms for small object detection [19–22]. Kaiming He proposed SPPNet [19] in 2014 to extract features of any aspect ratio region, which provides an idea for the detection algorithms such as YOLOv3 [23] and YOLOv4 [24]. FPN [20] is a multiscale feature fusion network structure. FPN combines high-level semantic features and low-level location features to effectively improve the detection accuracy of small targets. PANet [21] is an improved version of FPN, which adopts the top-down and bottom-up transmission mode to eliminate the problem of information loss from the bottom features to the high features. ASFF [22] is a novel feature fusion strategy, which reduces the conflict and inconsistency between different feature layers through adaptive spatial feature fusion and improves the effectiveness of feature pyramid.

In addition, some researchers [25, 26] try to add P6 and P7 detection layers after P5 with 32 times downsampling rate to improve the detection accuracy of small objects, but it brings huge computational cost and speed loss. YOLO series algorithm [13, 23, 24, 27] is one of the faster one-stage algorithms, especially the YOLOv4. It improves the low accuracy of YOLO [13], YOLOv2 [27], and YOLOv3 by combining the advantages of a large number of excellent models and adding a large number of training tricks. However, both YOLOv4 and previous algorithms are trained and optimized for MS-COCO [28], which requires a large number of categories to be detected and its context is highly variable. So these models are suboptimal when applied to the field of autonomous driving. Therefore, this paper proposes a new method to improve the accuracy of the model by embedding the RFB module [18] into the backbone network, optimizing the PAN, adding attention module CBAM [29] and CA [30], and reducing the computation, improving the real-time performance by scaling the width of the network.

## 2. Related Work

**Figure 1:**
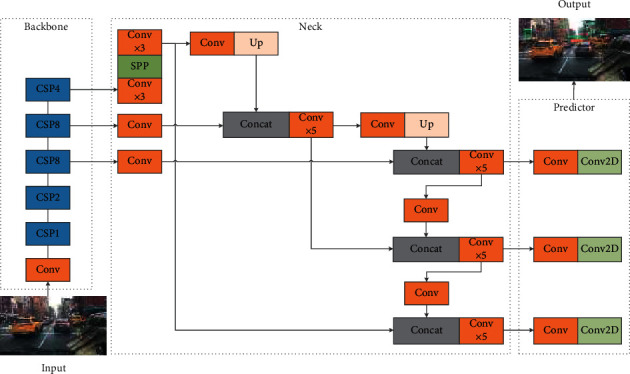
YOLOv4 structure.

YOLO [13] is different from the two-stage algorithm using region proposal to get regions of interest. Instead, it detects objects by segmenting the image into grid cells. Its output layer information includes bbox coordinates, confidence, and classification score. Therefore, it can detect multiple objects through a single stage, and the speed is much faster than two-stage algorithm. However, due to the fact that it predicts coordinates directly and not based on anchor, it is difficult to detect small objects. YOLOv2 [27] adds BN layer after convolution layer, applies the idea of bbox based on anchor, multiscale training, and uses passthrough layer to fuse fine-grained features, which improves the accuracy compared with YOLO and YOLOv3 [23]; its backbone DarkNet53 applies residual connection to solve the problem of deep network gradient disappear; FPN feature fusion retains small object fine-grained features; multiscale prediction makes the network detect objects of different sizes. It has a more obvious improvement compared with YOLO and YOLOv2. The structure of YOLOv4 [24] is shown in Figure 1. On the basis of YOLOv3, a large number of excellent methods and training tricks in recent years are tried. Backbone CSPDarkNet53 is DarkNet53 integrated into CSP structure [31]. The SPP module [19] after the backbone significantly increases the receptive field but hardly affects the inference speed. The repeated extraction process of PAN [21] structural features alleviates the problem of serious information loss when the bottom information is transferred to the top in FPN. As with YOLOv3, the prediction layer is carried out on three different scales to detect objects of different sizes. The inference speed of YOLOv4 is faster than that of YOLO and YOLOv2 because it only consists of 1 × 1 and 3 × 3 small convolution layers. The parameters of the backbone with CSP structure are greatly reduced, and the information exchange between layers is greatly improved. Therefore, the inference speed and accuracy are better than those of YOLOv3. It can also satisfy the high real-time requirement of autonomous driving system. However, generally speaking, its accuracy is still lower than that of the two-stage algorithm, and it does not optimize for the situation of many small objects in the autonomous driving scene. To make up for this, we use YOLOv4, which has a lower complexity than the two-stage algorithm, and improve the accuracy and speed of YOLOv4 through additional methods, so as to design a more efficient detector for autonomous driving.

Since SENet [32] shined in the last ImageNet classification competition in 2016, the attention module of plug-and-play can be directly applied to the existing neural network because of its flexibility, which is popular in computer vision tasks. CBAM [29] considers the location information ignored by SE module and uses large-scale convolution to utilize the location information by reducing the number of channels, which has better interpretability than SE module. CA [33] is a newly proposed attention module. In order to alleviate the loss of location information caused by 2D global pooling, channel attention is decomposed into two parallel 1D feature decoding processes, and the location information is effectively embedded into channel attention.

Traditional object detection algorithm usually uses mean square error (MSE, L2) or smooth L1 [9] to regress the center point coordinates and the width and height of bbox directly, i.e., {*x*_center_, *y*_center_, *w*, *h*}, or the upper left corner and lower right corner, i.e., {*x*_top left_, *y*_top left_, *x*_bottom right_, *y*_bottom right_}. For the anchor-based object detection algorithm, it is to regress the offset, that is, {*x*_offset_, *y*_offset_, *w*_offset_, *h*_offset_}. But regression of bbox directly is to take the four bbox points as independent variables, without considering the correlation between them, and in the process of training, it is more inclined to large objects, because the loss of small objects is originally small. Therefore, in order to better deal with this problem, IoU loss [34] was proposed to treat bbox as a whole regression and take GT into account. IoU has scale invariance; it can solve the problem that loss increases with scale in regression. Recently, with the continuous improvement of researchers, GIoU loss [30] was proposed. In addition to IoU, GIoU loss also considers the shape and direction of the object to solve the problem that IoU loss can not reflect the size of coincidence degree and return gradient when IoU is zero. DIoU loss [35] is to replace the penalty term of GIoU to maximize the overlap area with the minimum circumscribed rectangle by minimizing the Euclidean distance of bbox and GT center points, so as to accelerate the convergence. As for CIoU loss [35], the aspect ratio is considered on the basis of DIoU. This year, some researchers put forward EIoU loss [36], thinking about that the relative aspect ratio in CIoU loss cannot reflect the real difference with its confidence, so the real width loss and high loss are calculated, respectively, and then added up.

**Figure 2:**
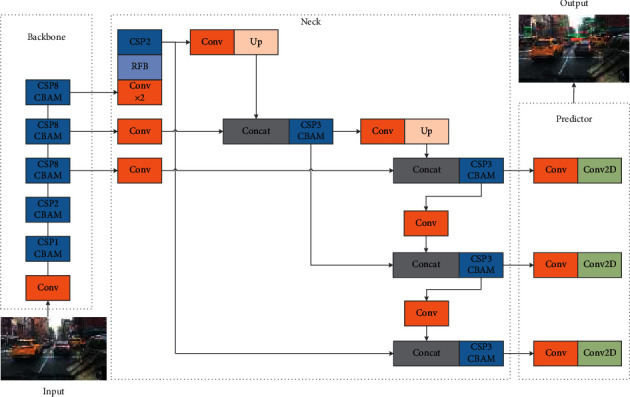
Proposed work (2) structure.

The autonomous driving scene is different from the daily life scene, which does not need to pay attention to those unimportant classes. Therefore, most of the advanced models optimized for MS-COCO [28] are suboptimal. KITTI [37] is a common dataset in autonomous driving scenes. It is collected in urban areas, rural areas, and expressways. Each image has up to 15 cars and more than 30 pedestrians, and there are various degrees of occlusion and truncation. BDD100k [38] is a large and diverse public driving dataset released by the Berkeley AI Research (BAIR) in recent years, including different weather conditions, day and night, as well as different lighting conditions and occlusion. This paper proposes two algorithms based on YOLOv4. The first algorithm improves the accuracy by adding CSP [31] structure into feature fusion, inserting attention mechanism, and using EIoU regression loss function to accelerate model convergence. The second algorithm improves the detection accuracy of dense small objects by inserting RFB [18] module. Finally, the width is reduced to 3/4 of the original to improve the inference speed, as shown in Figure 2.

## 3. Proposed Work

According to YOLOv4 [24], the anchor-based one-stage detection algorithm is generally composed of backbone, neck, and predictor head. The first model proposed in this paper inserts the attention mechanism into the bottleneck of the residual structure and adds the CSP structure into the neck as the baseline of this paper. In addition, in model 2, slightly adjust the number of iterations of the backbone, adjust the insertion position of the attention mechanism, replace the SPP structure, and scale the overall network structure in the width direction. The improved algorithm meets the needs of real-time detection. It is a multiscale real-time detection algorithm specially designed for autonomous driving scene.

### 3.1. Backbone

**Figure 3:**
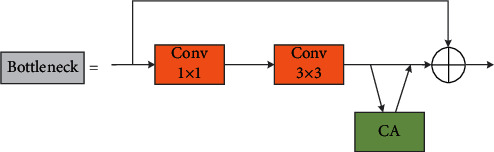
Coordinate attention in bottleneck.

**Figure 4:**
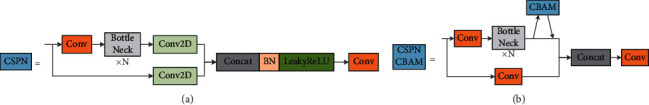
(a) CSP in YOLOv4. (b) CSP in proposed work (2).

CSPDarkNet53 of YOLOv4 is an excellent backbone, which can solve the task of feature extraction in most detection scenes. The first model proposed in this paper continues to use CSPDarkNet53 and only adds CA attention module into bottleneck (see Figure 3). The effectiveness of attention mechanism has been fully verified in many detection models. It can greatly increase the ability of feature extraction by adding only a small number of parameters. In order to more fully enhance the feature extraction ability of backbone in complex traffic scenes, the second model doubled the number of iterations of the last layer of its residual structure (i.e., increased to 8). In the experiment, it was found that it is better to modify the attention mechanism to CBAM and the insertion position to be outside the residual structure and inside the CSP structure, as shown in Figure 4(b).

**Figure 5:**
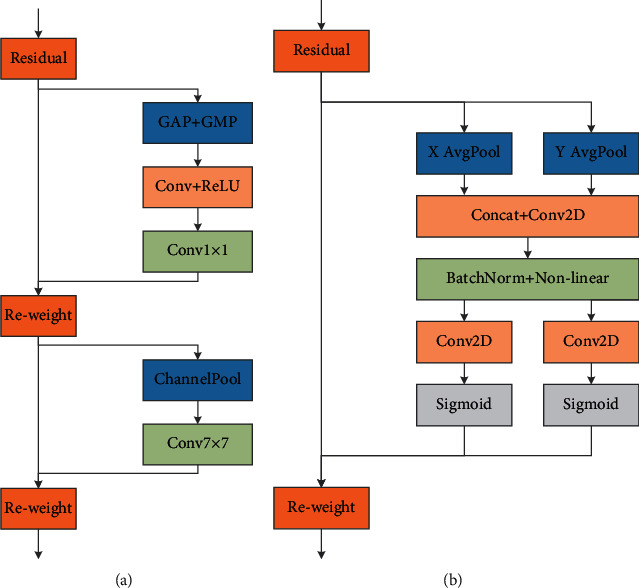
Attention mechanism. (a) CBAM. (b) CA.

CBAM [29] and CA [30] modules are shown in Figure 5. Both CBAM and CA are attention mechanisms of mixed channel and space. Compared with the single channel attention mechanism SE [32], the neural network will pay more attention to the object area containing important information, suppress irrelevant information, and improve the overall accuracy of object detection. Figure 3 is the CA attention mechanism insertion position of model 1.

### 3.2. Neck

**Figure 6:**
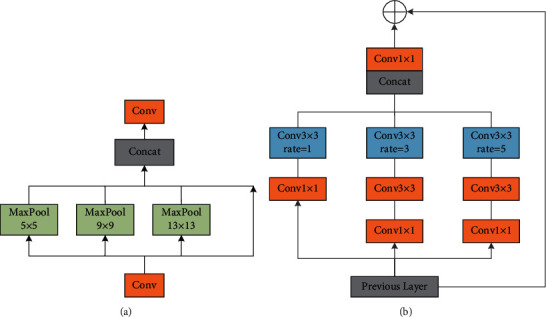
(a) SPP layer. (b) RFB layer.

For CNN, the more backward layers are rich in semantic information. YOLOv4 uses SPP [19] after backbone to increase the receptive field of the network. Compared with the pure pooling of SPP, RFB [18] draws lessons from Inception in structure, adopts the horizontal connection fusion network layer, and increases the receptive field and reduces the amount of calculation through dilated convolution, which is more robust. As shown in Figure 6, RFB block is composed of 3 × 3 convolution and three dilated convolution layers.

PAN [21] is a feature enhancement structure for feature fusion. It adopts a top-down and bottom-up transmission mode to eliminate the loss of feature information from the bottom feature to the high feature. However, the layer structure between PAN is connected in the form of ordinary convolution. CSP [31] structure has shown its advantages in backbone: strengthening information exchange between channels and reducing the amount of calculation. Therefore, adding CSP structure to the layer structure between PAN is more refined and has less parameters than CSP structure in CSPDarkNet53 (see Figure 4).

### 3.3. Predictor Head

In object detection, the conflict between classification and regression tasks is a well-known problem, so the prediction head for classification and regression is widely used in most detectors. YOLOv4 follows the predictor head of YOLOv3, which consists of one 3 × 3 and one 1 × 1 convolution layer. The final predicted output channel is *na* × (4+1+*nc*), where *na* is the number of anchors in each detection layer and *nc* is the number of classes. Proposed work follows this structure.

### 3.4. Loss Function

For the object detection model, the loss function is generally the sum of confidence loss, classification loss, and bbox regression loss. Binary cross entropy (BCE) was used for confidence loss and classification loss, and EIoU loss was used for bbox regression loss.

(1) $$\begin{array}{c} L=\lambda_{1}L_{\text{obj}}+\lambda_{2}L_{cls}+\lambda_{3}L_{\text{box}}, \end{array}$$

(2) $$\begin{array}{c} L_{\text{obj}}=-\frac{1}{N}\sum_{i}\left(O_{i}\ln\left(\hat{C_{i}}\right)+\left(1-O_{i}\right)\ln\left(1-\hat{C_{i}}\right)\right), \end{array}$$

(3) $$\begin{array}{c} L_{\text{cls}}=-\frac{1}{N_{\text{pos}}}\sum_{i\in \text{pos}}\sum_{j\in \text{cls}}O_{ij}\ln\left({\hat{C}}_{ij}\right)+\left(1-O_{ij}\right)\ln\left(1-{\hat{C}}_{ij}\right), \end{array}$$

(4) $$\begin{array}{c} L_{\text{box}}=L_{\text{EIoU}}=L_{\text{IoU}}+L_{\text{dis}}+L_{\text{asp}}=1-\text{IoU}+\frac{\rho^{2}\left(b,b^{gt}\right)}{C^{2}}+\frac{\rho^{2}\left(w,w^{gt}\right)}{C_{w}^{2}}+\frac{\rho^{2}\left(h,h^{gt}\right)}{C_{h}^{2}}. \end{array}$$

In formula (1), *λ*_1_, *λ*_2_, *λ*_3_ are the coefficient of each loss, which are hyperparameters. In formula (2) *O*_*i*_*ε*[0,1] represents the IoU of the predicted bounding box and the groud truth, $\hat{C_{i}}=\text{sigmoid}\left(C_{i}\right)$, *C*_*i*_ is the predicted value, and *N* is the number of positive and negative samples. In formula (3), *O*_*ij*_*ε*{0,1} indicates whether there is a *j*_th_ class in the *i*_th_ prediction bounding box, ${\hat{C}}_{ij}=\text{sigmoid}\left(C_{ij}\right)$, *C*_*ij*_ is the predicted value, and *N*_pos_ is the number of positive samples. In formula (4), *ρ*^2^(*b*, *b*^*gt*^) denotes the Euclidean distance between the center points of bbox and GT, *C* is the diagonal of the smallest circumscribed rectangle of the two boxes, and *C*_*w*_, *C*_*h*_ are the width and height of the minimum circumscribed rectangle.

### 3.5. The Performance of Different Models

The parameter quantity and calculation quantity of different network model weights are shown in Table 1. All models are tested at 512 × 512 resolution, with FP16-precision.

It can be seen that the parameters of proposed work (1) are 11.61M less than YOLOv4 and 6.35M less than YOLOv3. The parameters of proposed work (2) are reduced by 41.3% and 36.1%, respectively, compared with YOLOv4 and YOLOv3. In addition, from the perspective of FLOPs, proposed work greatly reduces the complexity. At the same time, in terms of model size, proposed work (2) only occupies 72.1 MB, which is 40.9% less than that of YOLOv4, which largely depends on the impact of CSP structure introduced in neck and 3/4 reduction in overall width. It is suitable for carrying and using in autonomous driving.

## 4. Experiment

### 4.1. Dataset

In the experiment, we used KITTI [37] and BDD100k [38], which are commonly used in autonomous driving research. KITTI dataset consists of 7481 training sets and 7518 test sets, including three classes: Car, Cyclist, and Pedestrian. Since the test set has no label, the training set and the validation set are split by randomly dividing the training set into two halves [39, 40]. BDD100k dataset is composed of 70,000 training sets, 10,000 validation sets, and 20,000 test sets, including ten classes: person, rider, car, bus, truck, bike, motor, traffic light, traffic sign, and train. The ratio of training set and verification set is 7 : 1. There are about 1.46 million object instances in training set and validation set, of which about 0.8 million are car instances, while only 151 are train instances. This kind of unbalanced distribution among categories will lead to the decline of network feature extraction ability, so train, rider, and motor are ignored in the final evaluation. The final BDD dataset includes seven classes: person, car, bus, truck, bike, traffic light, and traffic sign. Since we only studied the differences between models, 1/5 of the training set and validation set are randomly sampled as the final dataset. The experiment was carried out on Ubuntu 18.04, NVIDIA Quadro M4000, CUDA 10.1, and cuDNN v7.6.5. The inference speed is related to the hardware equipment. The inference test FPS in this paper is carried out on NVIDIA RTX 2080Ti.

### 4.2. Anchor Design

For the KITTI and BDD datasets used in this paper, we set the anchor box size to obtain accurate prediction results. The results obtained by *k*-means clustering algorithm are shown in Table 2.

### 4.3. Performance Evaluation of Proposed Work

In order to check the effectiveness of the improved YOLOv4 network, a comparative experiment is carried out between the original YOLOv4 model and the improved YOLOv4 model. Generally speaking, the test results can be divided into four categories: TP (True Positive) is the positive sample of correct prediction; FP (False Positive) is the positive sample of false prediction; TN (True Negative) is the negative sample of correct prediction; FN (False Negative) is the negative sample of false prediction. The confusion matrix is shown in Table 3.

The number of all positive samples predicted by the model is TP + FP, and the proportion of correct positive samples is called precision, as shown in formula (5). The number of all positive samples in the validation set is TP + FN, and the proportion of predicted positive samples is called recall, as shown in formula (6).

(5) $$\begin{array}{c} \text{Precision}=\frac{\text{TP}}{\text{TP}+\text{FP}}, \end{array}$$

(6) $$\begin{array}{c} \text{Recall}=\frac{\text{TP}}{\text{TP}+\text{FN}}. \end{array}$$

AP value is usually used as a criterion to evaluate the performance of object detection model. AP value is the area enclosed by P-R curve (with recall as *x* axis and accuracy as *y* axis). AP represents the accuracy of the model in a certain category; mAP represents the average accuracy of all categories, which can measure the performance of the model in all categories. mAP50 represents all mAP values with IoU of prediction box and GT greater than 0.5. As shown in formulas (7) and (8).

(7) $$\begin{array}{c} \text{AP}=\int_{0}^{1}P\left(R\right)\text{d}R, \end{array}$$

(8) $$\begin{array}{c} \text{mAP}=\frac{\sum_{i=1}^{N}{\text{AP}}_{i}}{N}. \end{array}$$

For KITTI [37] dataset, the IoU of Car is usually set to 0.7, and Cyclist and Pedestrian are set to 0.5, while for BDD dataset [38], the IoU of all classes is set to 0.5. In the training of YOLOv4 and proposed model 1, the batch size is set to 16, while in model 2, the batch size is set to 32, the learning rate is set to 0.003, and 300 epochs are trained.

In order to reflect the performance of the improved model entirely, the evaluation results are compared with other researches [14, 18, 39]. These experimental results are from [39], as shown in Table 4. These researches are not included in Table 5 as AP50 evaluation results of BDD dataset.

As shown in Table 4, the mAP of YOLOv4 in KITTI validation set is 86.43%, while the mAP of model 1 is 88.49%, and the detection accuracy is improved by 2.06%. The mAP of model 2 is 86.35%, which is 0.08% lower than that of YOLOv4, but its parameters and calculation are much less, and the inference speed is 6.33FPS higher. Table 5 shows the performance of each model in each class of BDD validation set. Compared with YOLOv4, the mAP of model 1 is increased by 2.95% and that of model 2 is increased by 1.73%. In addition, it can be seen that model 1 and model 2 significantly improve the detection accuracy of small objects such as traffic lights and traffic signs. For large objects such as cars and trucks, the detection accuracy of the improved model is almost the same as that of the original YOLOv4. From these results, it can be concluded that model 1 and model 2 can fully improve the detection accuracy of small objects without sacrificing the detection accuracy of large objects. It is worth mentioning that when the input size is increased to 704 × 704, the mAP reaches 61.34%, but it is the high precision obtained at the expense of speed.

**Figure 7:**
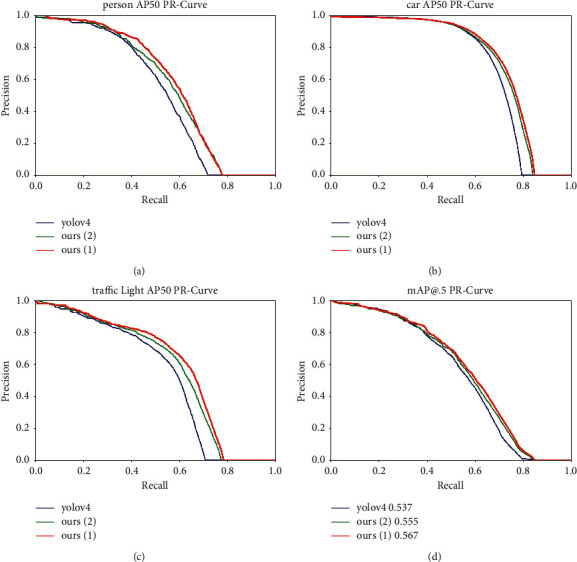
P-R curve.

In addition, the PR curves of the three common objects of the BDD dataset, cars, people, and traffic lights, are shown in Figure 7. PR curve is an important index for evaluating the output of object detection algorithm, and its area is the AP value of this class. It can be seen from Figure 7 that the PR curves of model 1 and model 2 completely surround the YOLOv4, which also shows the effectiveness of the proposed work.

### 4.4. Visual Evaluation

**Figure 8:**
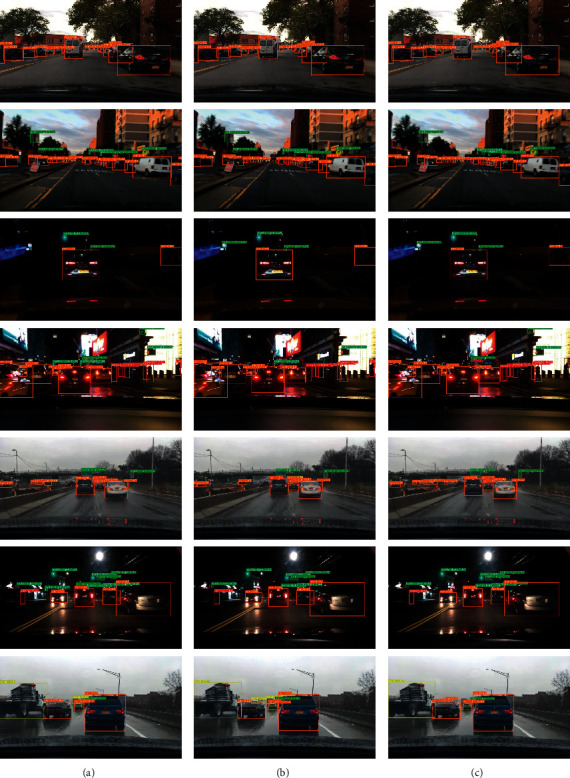
(a) YOLOv4 inference results. (b) Proposed work (1) inference. (c) Proposed work (2) inference.

Figure 8 shows the visual comparison of YOLOv4 and proposed work. It can be seen from the third row that, in the night environment, model 1 and model 2 can detect traffic light object missed by YOLOv4. In the fourth row, model 1 can supplement the detection of incorrect traffic sign in YOLOv4. In rows 5 and 6, model 1 and model 2 can find more small objects than YOLOv4. The weather in the first row and the last row is better, and the detection frame of the improved algorithm is more accurate.

Based on these results, model 1 and model 2 can significantly improve the detection accuracy, so as to improve driving stability and efficiency, prevent fatal accidents, meet the needs of autonomous driving real-time object detection task, and have practical application value.

## 5. Conclusions

Real-time object detection technology is of great significance in the field of autonomous driving. Aimed at the problem of insufficient accuracy of one-stage detector in autonomous driving scene, based on YOLOv4, this paper replaces SPP with RFB structure after backbone, integrates CSP structure with less computation into neck structure, and finally adds CBAM and CA attention mechanism to make the neural network pay more attention to the object area containing important information, suppress irrelevant information, and improve detection accuracy. The experimental results show that the improved model 1 has higher accuracy than the original YOLOv4 in object detection task. The mAP is improved by 2.06% in KITTI validation set and 2.95% in BDD validation set. The mAP50 of model 2 is increased by 1.73%, and the inference speed is increased by 4.83 fps, which verifies the effectiveness of the improved algorithm. It provides a theoretical reference for further practical application. In the follow-up work, some researchers are concerned about how to improve the detection accuracy of [7, 41, 42] at night and under bad weather conditions, and further improvement of the detection accuracy will also be our next research direction.

## Figures and Tables

**Table 1:** Comparison of proposed work and YOLOv4.

Models	Parameters (M)	GFLOPs	Model size (MB)
YOLOv3	58.70	100.1	117
YOLOv4	63.96	87.9	122
Proposed work (1)	52.35	71.3	100
Proposed work (2)	37.53	46.2	72.1

**Table 2:** *K*-means cluster.

	Anchor 1	Anchor 2	Anchor 3
KITTI			
Small object	(10,29)	(16,39)	(10,90)
Medium object	(24,53)	(37,71)	(27,197)
Large object	(57,101)	(79,163)	(129,246)

BDD			
Small object	(5,6)	(4,12)	(7,11)
Medium object	(6,20)	(13,17)	(10,37)
Large object	(22,30)	(41,57)	(99,136)

**Table 3:** Confusion matrix.

	Prediction	
Real	Positive	Negative
True	TP	TN
False	FP	FN

**Table 4:** Evaluation in KITTI.

Detection algorithm	Car AP70	Pedestrian AP50	Cyclist AP50	mAP (%)	FPS	Input size
MS-CNN [14]	87.42	80.43	86.28	84.71	8.13	1920 × 576
SINet [15]	89.82	79.20	87.23	85.42	23.98	1920 × 576
SSD [12]	85.12	48.06	50.68	61.28	28.93	512 × 512
RefineDet [17]	92.74	78.45	81.90	84.36	27.81	512 × 512
CFENet [16]	88.47	—	—	—	—	512 × 512
RFBNet [18]	86.39	61.62	72.31	73.44	39.20	512 × 512
YOLOv3 [23]	79.49	79.01	83.07	80.52	43.57	512 × 512
Gaussian YOLOv3 [39]	87.33	79.90	83.60	83.61	43.13	512 × 512
YOLOv4 [24]	90.50	80.10	88.70	86.43	52.14	512 × 512
Proposed work (1)	92.38	83.60	89.50	88.49	48.37	512 × 512
Proposed work (2)	90.05	81.10	87.90	86.35	58.47	512 × 512

**Table 5:** Evaluation in BDD.

Detection algorithm	Person	Car	Bus	Truck	Bike	Traffic light	Traffic sign	mAP50 (%)	FPS	Input size
YOLOv4	51.70	69.20	49.30	55.70	43.00	52.30	55.00	53.74	52.84	512 × 512
Proposed work (1)	57.30	73.00	50.20	54.00	43.50	58.90	59.90	56.69	48.56	512 × 512
Proposed work (2)	55.70	72.00	47.50	53.50	44.80	56.80	58.00	55.47	57.67	512 × 512
Proposed work (1)	54.90	77.60	54.90	59.40	50.40	65.30	66.90	61.34	41.20	704 × 704

## Data Availability

All data included in this study can be downloaded from the official websites of KITTI and BDD100k or obtained by contacting the corresponding authors.
